# Autologous Stem Cell Transplantation in Common Variable Immunodeficiency: A Case of Successful Treatment of Severe Refractory Autoimmune Encephalitis

**DOI:** 10.3389/fimmu.2020.01317

**Published:** 2020-06-25

**Authors:** Matthias Froehlich, Eva C. Schwaneck, Michael Gernert, Ottar Gadeholt, Patrick-Pascal Strunz, Henner Morbach, Hans-Peter Tony, Marc Schmalzing

**Affiliations:** ^1^Schwerpunkt Rheumatologie/Klinische Immunologie, Medizinische Klinik und Poliklinik II, Universität Würzburg, Würzburg, Germany; ^2^Rheumatologische Schwerpunktpraxis Würzburg, Würzburg, Germany; ^3^Kinderklinik und Poliklinik, Universität Würzburg, Würzburg, Germany

**Keywords:** common variable immunodeficiency, primary immunodeficiencies, autoimmunity, autologous stem cell transplantation, autoimmune encephalitis

## Abstract

Common variable immunodeficiency (CVID) is the most common primary immunodeficiency in adults. It is associated with hypogammaglobulinemia, recurring infections and autoimmune phenomena. Treatment includes immunoglobulin substitution and immunosuppressants. Autoimmune neurological manifestations of CVID are rare and occur predominantly as granulomatous disease. We report the case of a 35-year-old woman with CVID who developed autoimmune encephalitis as demonstrated by double cerebral biopsy. Infectious or malignant causes could be excluded. Despite intensive immunosuppressive therapy with common regimens no significant improvement could be achieved. Ultimately, an autologous hematopoietic stem cell transplantation (HSCT) was performed, resulting in lasting complete remission of the encephalitis. To our knowledge, this is the first report of refractory autoimmune phenomena in CVID treated by autologous HSCT.

## Introduction

Common variable immunodeficiency (CVID) is the most common primary immunodeficiency in adults. The primary finding is hypogammaglobulinemia (1). Clinical symptoms are heterogeneous with different levels of immune dysregulation (2). In addition to infectious complications, autoimmune manifestations, including immune cytopenias, pneumonia, inflammatory bowel disease, and granulomatous inflammation, occur in about 20% of cases (3). Central nervous system (CNS) involvement is rare in CVID; most data are found for cerebral granulomatous disease (4), one case reported unilateral optic neuritis (5). Management of CVID includes immunoglobulin replacement (IgRT), immunosuppressive therapy for autoimmune manifestations, and close surveillance for the development of additional comorbidities (2). In this case report, we present a young woman with CVID who developed autoimmune CNS involvement, comprising brain and spinal cord. To our knowledge, this is one of very few cases reporting non-granulomatous CNS involvement. In addition, this is the first case demonstrating the effective and safe performance of autologous HSCT as treatment of a severe, organ-threatening, refractory autoimmune manifestation in CVID.

## Case Presentation

A 35-year-old woman was admitted at our hospital for pleural empyema. Primary antibiotic treatment was followed by surgical removal of the affected lung sub-segment. Histology showed a fibrosing reaction with histological pattern of non-specific interstitial pneumonia (NSIP), as well as typical infectious features. Since adolescence, the patient suffered from recurring respiratory infections. At the age of 15, she developed immune thrombocytopenia, which was successfully treated with several cycles of intravenous immunoglobulins. In her early 30s, she twice suffered from herpes zoster reactivation.

Further examination revealed splenomegaly, abdominal lymphadenopathy, and decreased serum immunoglobulin levels. According to the guidelines of the European Society for Immunodeficiencies (ESID) (6), diagnosis of CVID could be made. Total immunoglobulin values at diagnosis were IgG 598 mg/dl, IgA < 5 mg/dl, IgM 27 mg/dl. The lymphocyte count was reduced (760/μl) with low levels of CD4+ T-helper cells (234/μl), reduced naïve CD4+ T-helper cells (11,2% of all CD4+ T-cells), but immunophenotyping showed a normal percentage of NK cells, T-cells and B-lymphocytes with disturbed maturation and reduction of switched memory B-cells and an increase in CD21 low B-cells, which according to the classification for immunodeficiencies (EUROclass) corresponds to the following subgroup: smB- TRhigh CD21low (7). Due to the low T-cell count, classification as a combined Immunodeficiency (CID) would also have been possible. Furthermore the patient displayed a decreased frequency of regulatory T cells (Treg) which also indicated a dysfunctional phenotype with low expression of CTLA4 (cytotoxic T-lymphocyte-associated Protein 4) as well as FOXP3 (Supplemental Figure 1). Molecular genetic testing for typical genetic defects in CVID such as LRBA, CD3G, IL2RA, LAT, LCK, PIK3CD, PIK3R1, PTEN, STAT3, ZAP70, or CTLA4 deficiency, yielded no results. Other causes of secondary hypogammaglobulinemia, such as HIV, were excluded. No lymphoma was found by bone marrow trephine biopsy or total body CT scan.

The patient recovered well under antibiotic therapy. Immunoglobulin replacement therapy (IgRT) with subcutaneous immunoglubulins (0.5 g/kg body weight every 4 weeks combined with hyaluronidase, target trough level of 6 g/l) was initiated. Despite stable clinical representation, a chest CT scan 2 months later showed progressive infiltrates of the lung parenchyma, as well as bronchiectasis. To rule out a new infection, a bronchoscopy was performed, which showed no evidence of bacterial or mycotic infection, including TB. Virus PCR for EBV, CMV, and common respiratory tract infections was negative.

We therefore considered the infiltrates a manifestation of CVID, most likely as granulomatous-lymphocytic interstitial lung disease (GLILD), and started immunosuppressive therapy with prednisolone (1 mg/kg) and subsequent taper, and azathioprine (2.5 mg/kg/day). A CT scan 6 months later showed a significant improvement of the pulmonary infiltrates, the patient had no relevant infections since starting IgG substitution.

**Figure 1:**
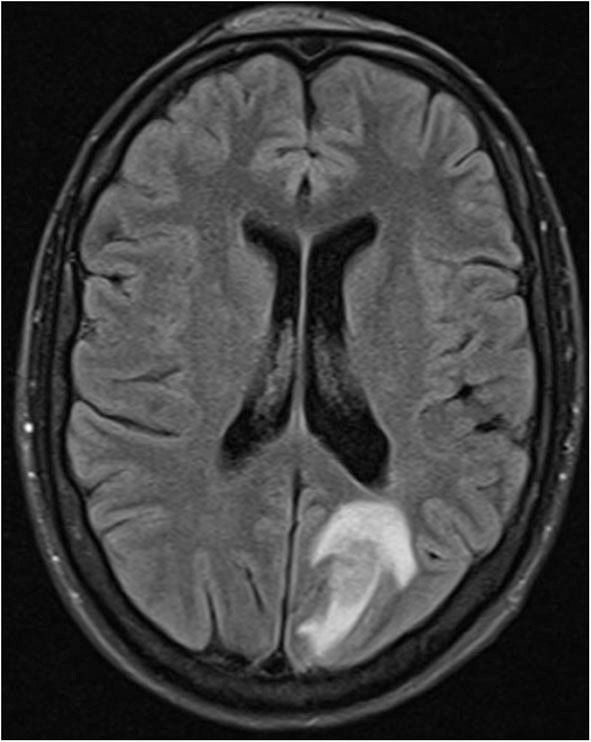
Hyperintense lesion with perifocal edema left occipital in fluid-suppressed T2 MRI technique (Fluid-attenuated inversion recovery (FLAIR) sequence).

4 months later the patient suffered a generalized epileptic seizure. Cerebral MRI showed several periventricular, subependymal, and leptomeningeal lesions with intensive contrast medium uptake. The criteria for multiple sclerosis were not met on the basis of the distribution pattern and a pattern of different ages of lesions. The largest lesion was located in the left posterior lobe with a diameter of 4.4 cm (Figure 1). CSF analysis showed a mild pleocytosis with a normal protein content. Oligoclonal bands were not detectable. A eubacterial 16S rRNA PCR was negative, as were PCRs for HSV, VZV, EBV, CMV, HHV6, HHV7, HHV8, adenovirus, enterovirus, BK virus, and JC virus.

We proceeded to biopsy the occipital lesion by stereotactic puncture. The histological examination showed an inflammation with predominantly perivascular accumulation of T-lymphocytes and an increase in plasma cells without kappa/lambda light chain restriction. Malignant cells, granulomas, or demyelinating plaques were not detected. Hence, the etiology remained unclear. Another whole-body CT scan as well as a further bone marrow trephine biopsy did not show any pathological findings. Due to the lack of clarity and the therapeutic relevance, a second stereotactic biopsy was performed. This second tissue sample was sent to the German Reference Laboratory for Neuropathology. Again, no signs of malignancy were found, as well as no evidence of infection or demyelination, the pattern of T-cell predominant perivascular lymphocytic infiltration was confirmed. Thus, the histological findings as well as the other previous findings were compatible with an autoimmune encephalitis. Important differential diagnoses like lymphoma or multiple sclerosis were excluded.

**Figure 2:**
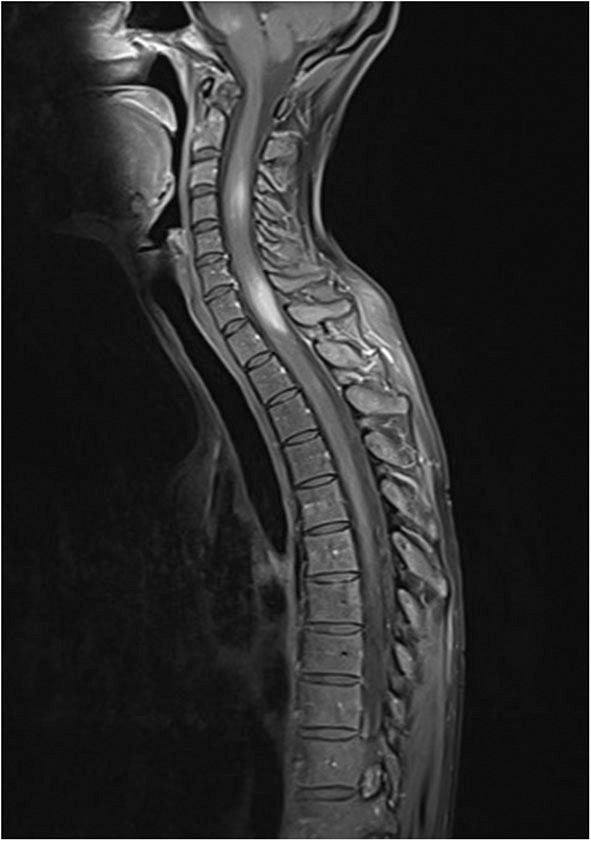
Hyperintense lesions in medulla oblongata and in cervical and upper thoracic spinal cord in contrast enhanced, fat suppressed T1 MRI technique.

In addition to IgRT, we started a more intensive immunosuppressive therapy with 2 cycles of rituximab 1,000 mg i.v. and high-dose steroids (prednisolone ~1.5 mg/kg/day) with subsequent taper. An MRI control of the CNS a few weeks later showed receding lesions. The patient's condition was stable with additional administration of an anticonvulsant and azathioprine as maintenance therapy. However, under the dose of azathioprine 2.5 mg/kg/day and prednisolone 15 mg/day, another MRI control 2 months later showed a renewed increase in intracerebral inflammatory activity with progressing lesions. Immunosuppressive therapy was escalated using cyclophosphamide (750 mg/m^2^ iv every 3 weeks), with additional trimethoprim/sulfamethoxazole prophylaxis. Azathioprine was discontinued. A staging MRI 3 cycles of cyclophosphamide showed a mixed response. New lesions were found in the medulla oblongata and in the posterolateral cervical medulla. MRI of the entire spinal cord showed focal lesions in the cervical and thoracic myelon extending to the Th2 segment (Figure 2). The clinical examination was inconspicuous, with no evidence of neurological symptoms.

We initiated therapy with abatacept, a T cell activation modulator 14 mg/kg bw i.v. (week 0, 2, then every 4 weeks), also due to the known Treg CTLA4 deficiency. In addition, a high-dose therapy with steroids (prednisolone 1,000 mg for 3 days) with subsequent dose reduction to 1 mg/kg body weight and then further tapering was performed. Again, MRI control of the CNS and spinal cord after initial improvement showed progression 3 months after starting abatacept.

Up to this point, no treatment regime had led to a sustained improvement in cerebral or spinal inflammation. The diagnosis underwent a critical review, including renewed CT, CSF analysis, and infection screening. No new findings were made. A further biopsy was not performed.

Due to the organ-threatening character of the inflammatory activity, which could not be controlled despite the previous intensive immunosuppression, we saw an autologous stem cell transplantation as the best possibility to intensify the therapy. Other therapy options did not seem promising in this situation. We used a well-established protocol according to the guidelines of European Group for Blood and Marrow Transplantation (EBMT). For mobilization of autologous hematopoietic stem cells, the patient received cyclophosphamide 2 g/m^2^ together with a daily dose of 105 μg G-CSF, starting on the second day after cyclophosphamide administration. Leukapharesis was performed on day 10. The autologous hematopoietic stem cells underwent CD34+-selection using immunomagnetic separation (CliniMACS CD34 Complete Kit, Miltenyi Biotec, Bergisch Gladbach, Germany). 8 weeks after apheresis, conditioning with cyclophosphamide (4 × 50 mg/kg body weight) and rabbit antithymocyte globulin (rATG) (3 × 5 mg/kg body weight) was administered over 5 days. On day 1 after conditioning, the autologous graft was transplanted (2.6 × 10^6^ CD34+ cells/kg body weight). During the subsequent aplasia phase until engraftment, the patient had several typical adverse events. Due to thrombocytopenia a small, non-significant subdural hematoma occurred. Substitution of 2 platelet concentrates stabilized hemostasis sufficiently. In addition, 2 red cell concentrates were given due to severe anemia. Apart from sinusitis, which was treated with antibiotics, no relevant infectious complications occurred. The patient received oral acyclovir and posaconazole prophylaxis. IgRT was continued unchanged to keep immunoglobulin levels stable within target range (6 g/l). Recovery of neutrophils above 500/μl occurred on day 14 after transplantation. Lymphocyte counts remained low at 700/μl on day 30. However, rather low values had already been measured before transplantation, probably because of the CVID itself, or due to immunosuppressive therapy. As was to be expected, the number of CD4+ T-helper cells was significantly reduced after transplantation (52/μl on day). On the day of discharge a low dose of prednisolone (10 mg/day) was maintained, as well as prophylactic oral therapy with trimethoprim/sulfamethoxazole, oral amphotericin B, and acyclovir. A CNS-MRI 3 weeks after discharge showed a significant decrease in intracerebral and intraspinal inflammation. Further controls after 3, 7, and 12 months showed a complete disappearance of the lesions. The health condition of the patient improved steadily. Regular controls of the immune reconstitution by immunophenotyping showed a gradual increase of T cells and B cells in the following months. After 11 months the CD4+ T helper cells reached 200/μl, so that acyclovir and amphotericin B were discontinued, trimethoprim/sulfamethoxazole was continued. As a sign of increased plasma cell activity during reconstitution there was an increase in polyclonal IgM. We decided to administer rituximab as B cell depleting maintenance therapy (1,000 mg, day 0, 15). The first cycle was given 9 months after transplantation, a second cycle 6 months later, resulting in a complete depletion of B cells and a decrease in IgM. Prednisolone was reduced to a minimal dose of 2.5 mg/day. 18 months after transplantation the patient is in good health without autoimmune symptoms, regular MRI controls show a sustained remission. Under persistent IgRT no severe infection has occurred since transplantation. The patient resumed her daily activities and her former profession.

## Discussion

We present a case of a female patient (35) with autoimmune encephalitis as a CNS manifestation of CVID, which was successfully treated by immunoablative conditioning and transplantation of autologous CD34-selected stem cells. CNS involvement in CVID is rare, especially in the form of autoimmune encephalitis (4). It is therefore of utmost importance to distinguish between an autoimmune CVID manifestation of the CNS and differential diagnoses, especially cerebral lymphoma or infectious complications, as far as possible. This was done in our patient by repeated imaging, two biopsies and extensive testing of CSF.

There is increasing evidence that, in addition to B cell dysfunction, T cell dysfunction plays an important role in autoimmunity in CVID (8). In a large cohort, it was shown that total T cells in CVID patients with autoimmune symptoms were lower than in those without autoimmunity (9). The degree of reduction of CD4+ T cells correlates with the severity of autoimmune symptoms (10, 11). Within the CD4+ T cells, a reduced number of regulatory T cells is associated with autoimmunity in CVID (12). Our patient showed normal total T cells, however, a reduced expression of the T cell surface protein CTLA4 on T reg could be detected as surrogate of disturbed T reg function (13). Mutations of CTLA4 are common in CVID patients and are associated with autoimmunity through the disruption of self-tolerance regulation (11). Based on these findings, our patient was treated with abatacept, a T-cell activation modulator, which has been shown to be effective in the treatment of other syndromes associated with CTLA4 deficiency (14). In this patient, abatacept was ineffective.

The most intensive form of therapy for primary immunodeficiencies (PID) is allogenic stem cell transplantation. Since most forms of PID are based on monogenetic defects intrinsic to hematopoietic cells (15), the treatment of primary immunodeficiency by replacing the mutated cells with healthy donor hematopoietic stem cells and establishing alloimmunity is a potentially curative approach. Based on proper strategies in selection of a suitable donor (16–19) and GvHD prophylaxis, reduced intensity conditioning regimes (20) and better supportive care overall survival reaches up to 85% today even for patients who undergo transplantation in young adulthood (21).

The role of allogeneic stem cell transplantation in CVID is controversial. Wehr et al. (22) showed results of a retrospective evaluation of 25 patients who underwent allogeneic stem cell transplantation for CVID. The indication was mainly based on the presence of immune dysregulation and not on infections. However, the mortality rate of CVID patients was 52% after allogeneic HSCT, which is significantly higher than for other PID. The main causes were treatment-resistant graft-vs.-host disease and infectious complications. On the other hand, survivors no longer needed IgRT in 50% of cases, and in 92% of surviving patients, health complaints that were the indication for allogeneic stem cell transplantation were significantly improved.

Autologous stem cell transplantation aims to “reset” the immune system by eradicating the autoreactive immunological memory. In contrast to allogeneic stem cell transplantation, transfusion of the patient's own stem cells does not generate alloimmunity. Immuno-ablative therapy and transfusion of CD34-selected stem cells causes a profound regeneration of the adaptive immune system with lasting changes in T-cell and B-cell subpopulations from memory to naive cell dominance (23, 24). The transplantation leads to a renewal of naive T cells including regulatory T cells via reactivation of the thymus (25). Furthermore, a diversification of the T cell receptor repertoire can be observed (26, 27). This can only be partially seen in our patient so far: While there was a normalization of natural killer cells within 6 months after transplantation, a persistent reduction of T cells was also detectable 18 months after the procedure with low levels of naïve CD4+ T cells. Delayed repopulation of naive CD4+ T lymphocytes was seen in several studies up to 24 months after transplantation (28–30) and correlated with an increase in TCR excision circles (TREC) (31, 32), which serve as a surrogate for increased biosynthesis of the T cell receptor (33, 34).

In this context it could also be shown that TREC levels were higher in autologous stem cell transplantation when a CD34+ selection of the transplant was performed (25). However, the role of CD34+ selection in SCT of autoimmune diseases is controversially discussed. A retrospective analysis of autoimmune patients in the European Bone Marrow Transplantation Database (EBMT) did not show an improvement of response using CD34+ selected grafts (35). A randomized study on CD34+ selection in autologous stem cell transplantation in patients with rheumatoid arthritis did not show any benefit in the outcome (36), nor did a recent retrospective study evaluating CD34+ selection in systemic sclerosis (37). On the other hand, it is considered that the return of a lymphocyte-depleted transplant results in the most complete eradication of autoreactive T cell clones, which is the basis for a stable, long-lasting remission. This hypothesis is supported by studies in multiple sclerosis patients who received a CD34+ selected autologous stem cell transplant and subsequently had no evidence of new inflammatory activity for up to 13 years after treatment (38). In addition, a prospective evaluation of the EBMT on patients with systemic sclerosis could now demonstrate a benefit of CD34+ selection in autologous stem cell transplantation in terms of outcome, which could possibly be due to the now more homogeneous conditioning regimen (39). A Japanese study recently showed similar results (40).

One of the main indications for autologous stem cell transplantation in autoimmune diseases today is multiple sclerosis (MS) (35), which is also an autoimmune cerebral inflammation. In contrast to CVID, several studies have already well investigated the effectiveness of autologous stem cell transplantation on MS (41). In a meta-analysis of 280 patients who received autologous stem cell transplantation, the overall survival rate was 93% after 5 years (42). Depending on the different subtypes of the disease, a progression-free survival in terms of a deterioration of the Expanded Disability Status Scale (EDSS) of up to 73% after 5 years was achieved. Similar to the analysis of Wehr et al. on allogeneic transplantation, a higher age, the number of previous therapies and the intensity of the conditioning regimen were predictors of a worse outcome. Despite several pre-treatments, the good response of our patient is thus possibly favored by the young age and the only minor chronic organ damage.

Taken together, good results on the effectiveness in refractory autoimmune diseases, and the excellent control of infectious risk by IgRT in this patient, made us favor autologous against allogeneic SCT as a treatment option.

So far, there are no guidelines for the therapeutic procedure after successful transplantation of autoimmune diseases, the role of immunosuppressive maintenance therapy was not examined in studies yet and remains controversial. In our patient, IgRT was continued unchanged. Maintenance treatment with rituximab was established due to possibly severe consequences of a relapse and signs of transient excessive B cell activation after SCT.

## Conclusion

Autologous hematopoietic stem cell transplantation can be a highly effective therapy for the treatment of severe refractory autoimmune manifestations of CVID. In the present case, it has been shown to induce sustained remission in the rare and life-threatening case of autoimmune encephalitis. Since autoimmunity is also the indication for transplantation in most cases in allogeneic stem cell transplantation in CVID, autologous transplantation could be a viable alternative for the treatment of these patients, considering the high periprocedural morbidity and mortality of allogeneic transplantation in CVID to date. However, the management of the therapy requires a high level of expertise, and studies on larger collectives would therefore be highly desirable.

## Data Availability Statement

All datasets generated for this study are included in the article/Supplementary Material.

## Ethics Statement

Written informed consent was obtained from the patient for the publication of any potentially identifiable images or data included in this article.

## Author Contributions

All authors were involved in drafting the article or revising it critically for important intellectual content, and all authors approved the final version to be submitted for publication. MF had full access to all of the data in the Case Report and takes responsibility for the integrity of the data.

## Conflict of Interest

The authors declare that the research was conducted in the absence of any commercial or financial relationships that could be construed as a potential conflict of interest.

## Supplementary Material

Supplemental Figure 1 Disturbed phenotype of regulatory T cells. Frequency of CD25+CD127-FOXP3 regulatory T cells (Treg) within CD4+ T cells **(A)**, fluorescence intensity (MFI) of FOXP3 expression in CD25+CD127-CD4+ T cells **(B)**, as well as ratio of CTLA4 expression between CD45RO+ Treg and CD45RO-CD25+/-CD127+FOXP3- naïve conventional T cells (Tcon) of healthy control individuals (Co) and the patient **(C)**. The 10th and 90th percentile of each parameter within the group of control indivdiuals is shown as dashed lines. The expression of FOXP3 in CD4+CD25+CD127- T cells of the patient and a healthy control is shown in **(D)**. The expression of CTLA-4 in CD45RO+ Treg and CD45RO- Tcon of the patient and a healthy control is shown in **(E)**.
