# Supplementary figures and images for: Autologous Stem Cell Transplantation in Common Variable Immunodeficiency: A Case of Successful Treatment of Severe Refractory Autoimmune Encephalitis
Source: Front Immunol. 2020 Jun 25;11:1317. doi: 10.3389/fimmu.2020.01317 (PMC7330058; doi:10.3389/fimmu.2020.01317)

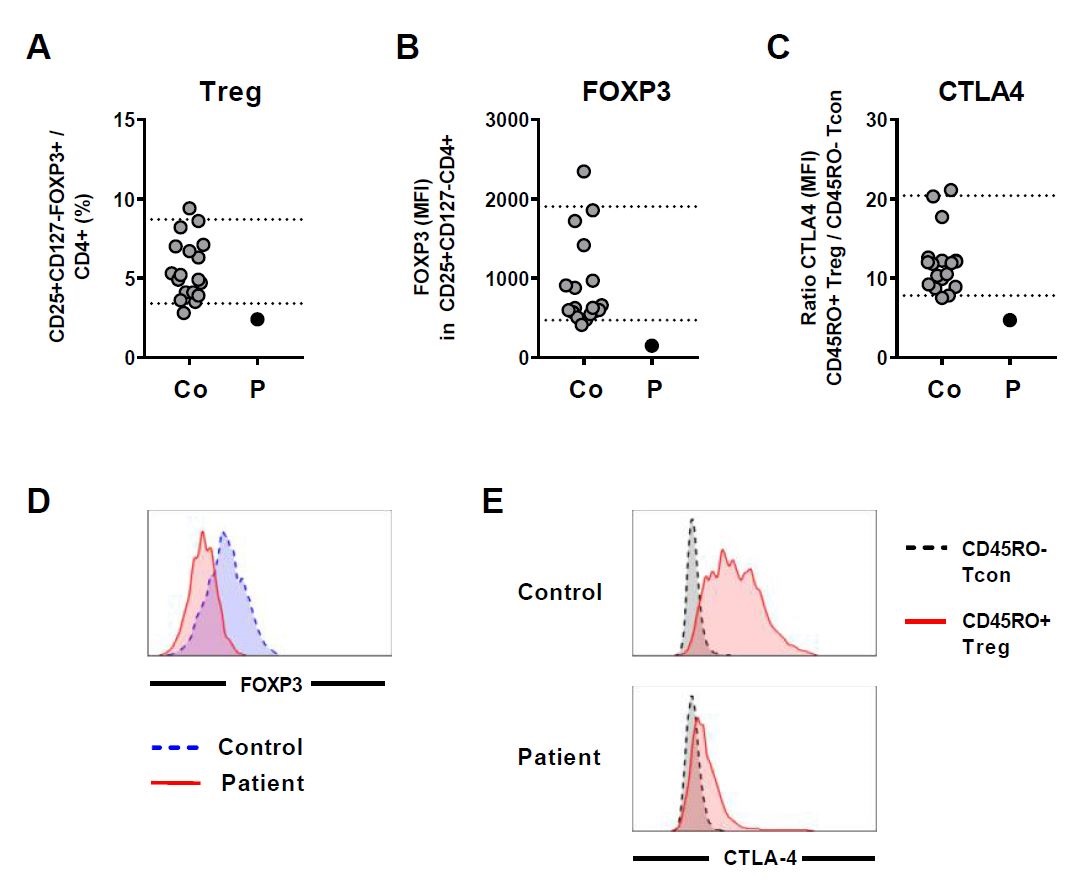

Supplement: Supplemental Figure 1 — Disturbed phenotype of regulatory T cells. Frequency of CD25+CD127-FOXP3 regulatory T cells (Treg) within CD4+ T cells (A), fluorescence intensity (MFI) of FOXP3 expression in CD25+CD127-CD4+ T cells (B), as well as ratio of CTLA4 expression between CD45RO+ Treg and CD45RO-CD25+/-CD127+FOXP3- naïve conventional T cells (Tcon) of healthy control individuals (Co) and the patient (C). The 10th and 90th percentile of each parameter within the group of control indivdiuals is shown as dashed lines. The expression of FOXP3 in CD4+CD25+CD127- T cells of the patient and a healthy control is shown in (D). The expression of CTLA-4 in CD45RO+ Treg and CD45RO- Tcon of the patient and a healthy control is shown in (E). [file Image_1.JPEG]
